# Japanese POEMS syndrome with Thalidomide (J-POST) Trial: study protocol for a phase II/III multicentre, randomised, double-blind, placebo-controlled trial

**DOI:** 10.1136/bmjopen-2014-007330

**Published:** 2015-01-08

**Authors:** Kanako Katayama, Sonoko Misawa, Yasunori Sato, Gen Sobue, Ichiro Yabe, Osamu Watanabe, Masatoyo Nishizawa, Susumu Kusunoki, Seiji Kikuchi, Ichiro Nakashima, Shu-ichi Ikeda, Nobuo Kohara, Takashi Kanda, Jun-ichi Kira, Hideki Hanaoka, Satoshi Kuwabara

**Affiliations:** 1 Clinical Research Center, Chiba University Hospital, Chiba, Japan; 2 Department of Neurology, Graduate School of Medicine, Chiba University, Chiba, Japan; 3 Department of Neurology, Nagoya University Graduate School of Medicine, Nagoya, Japan; 4 Department of Neurology, Hokkaido University Graduate School of Medicine, Sapporo, Japan; 5 Department of Neurology and Geriatrics, Kagoshima University Graduate School of Medical and Dental Sciences, Kagoshima, Japan; 6 Department of Neurology, Brain Research Institute, Niigata University, Niigata, Japan; 7 Department of Neurology, Faculty of Medicine, Kinki University, Osaka-Sayama, Japan; 8 Department of Neurology, National Hospital Organization Hokkaido Medical Center, Sapporo, Japan; 9 Department of Neurology, Tohoku University Graduate School of Medicine, Sendai, Japan; 10 Department of Medicine (Neurology and Rheumatology), Shinshu University School of Medicine, Matsumoto, Japan; 11 Department of Neurology, Kobe City Medical Center General Hospital, Kobe, Japan; 12 Department of Neurology and Clinical Neuroscience, Graduate School of Medicine Yamaguchi University, Ube, Japan; 13 Department of Neurology, Neurological Institute, Graduate School of Medical Sciences, Kyushu University, Fukuoka, Japan

## Abstract

**Introduction:**

Polyneuropathy, organomegaly, endocrinopathy, M-protein and skin changes (POEMS) syndrome is a fatal systemic disorder associated with plasma cell dyscrasia and the overproduction of the vascular endothelial growth factor (VEGF). Recently, the prognosis of POEMS was substantially improved by introduction of therapeutic intervention for myeloma. However, no randomised clinical trial has been performed because of the rarity and severity of the disease.

**Methods and analysis:**

The Japanese POEMS syndrome with Thalidomide (J-POST) Trial is a phase II/III multicentre, double-blinded, randomised, controlled trial that aims to evaluate the efficacy and safety of a 24-week treatment with thalidomide in POEMS syndrome, with an additional 48-week open-label safety study. Adults with POEMS syndrome who have no indication for transplantation are assessed for eligibility at 12 tertiary neurology centres in Japan. Patients who satisfy the eligibility criteria are randomised (1:1) to receive thalidomide (100–300 mg daily) plus dexamethasone (12 mg/m^2^ on days 1–4 of a 28-day cycle) or placebo plus dexamethasone. Both treatments were administered for 24 weeks (six cycles; randomised comparative study period). Patients who complete the randomised study period or show subacute deterioration during the randomised period participate in the subsequent 48-week open-label safety study (long-term safety period). The primary end point of the study is the reduction rate of serum VEGF levels at 24 weeks.

**Ethics and dissemination:**

The protocol was approved by the Institutional Review Board of each hospital. The trial was notified and registered at the Pharmaceutical and Medical Devices Agency, Japan (No. 22-1716). The J-POST Trial is currently ongoing and is due to finish in August 2015. The findings of this trial will be disseminated through peer-reviewed publications and conference presentations and will also be disseminated to participants.

**Trial registration number:**

UMIN000004179 and JMA-IIA00046.

**Strengths and limitations of this study**

- This study is the first randomised control trial for POEMS (polyneuropathy, organomegaly, endocrinopathy, M-protein and skin changes) syndrome and provides a major turning point in its therapeutic approach, as there is no other randomised or non-randomised controlled trial because of the rarity and severity of the disease.
- This trial will include patients with POEMS syndrome who represent close to 10% of the entire Japanese patient population; thus, the results are generalisable.
- This placebo-controlled trial can evaluate the efficacy and safety of thalidomide without biases.
- The natural history of the disease remains partially unclear.
- This trial employs a surrogate instead of a hard end point, which is the reduction rate of serum vascular endothelial growth factor levels over 24 weeks, as the primary end point; the adequacy of the surrogate end point should be validated in this study and future trials.

## Introduction

Polyneuropathy, organomegaly, endocrinopathy, M-protein and skin changes (POEMS) syndrome is a rare paraneoplastic disorder characterised by POEMS.^1^ A Japanese national survey conducted in 2003 showed that its prevalence is 0.3/100 000 population.^2^ Although the pathophysiology of POEMS remains unclear, plasma cell dyscrasia and the related overproduction of the vascular endothelial growth factor (VEGF) are assumed to play a central role in the disorder.^3^ ^4^ Moreover, VEGF levels are characteristically elevated in POEMS.^3^ ^5^ ^6^ VEGF levels were used recently as surrogate markers to evaluate disease activity,^7–10^ because it sometimes takes several years to evaluate therapeutic effects in POEMS syndrome on the basis of hard end points, such as relapse-free survival or overall survival.^10^ ^11^

The prognosis of POEMS syndrome was poor in the 1980s.^12^ ^13^ A large retrospective cohort study conducted in Japan reported that 38 of 58 patients who were treated mainly with corticosteroids died after a mean survival period of 33 months.^12^ Since around 2000, the prognosis of POEMS has been considerably improved by the successful application of treatments for multiple myeloma, such as high-dose chemotherapy with autologous stem cell transplantation (HDCT with ASCT) or immunomodulatory drugs.^7–9^ ^11^ ^14^ Currently, the therapeutic algorithm is the use of HDCT with ASCT as the first-line therapy, whereas patients who are not suitable for transplantation are treated with thalidomide or lenalidomide with dexamethasone. However, there is no established evidence of the efficacy of the new therapeutic interventions for POEMS, because the literature on these treatments includes only retrospective case reports or case series,^15^ or open single-arm study,^16^ because of the rarity and severity of the disease.

In addition, thalidomide, which is one of the standard treatment options for multiple myeloma, can suppress VEGF production and tumour proliferation.^17^ Previous case reports or case series reported that thalidomide improved or stabilised the clinical symptoms in patients with POEMS syndrome and decreased serum VEGF levels,^8^ ^18^ ^19^ and that it could be safely administered to patients who were not eligible for HDCT with ASCT because of older age or poor condition. However, randomised clinical trials are essential to investigate the efficacy and safety of new therapeutic interventions and to establish evidence and logical therapeutic strategies. Therefore, we designed the Japanese POEMS Syndrome with Thalidomide (J-POST) Trial, which is a phase II/III multicentre, double-blinded, randomised, controlled trial that aims to compare the efficacy and safety of a 24-week treatment with thalidomide with that of a placebo in POEMS syndrome, followed by a 48-week open-label safety study.

### Objectives

We examined the hypothesis that POEMS syndrome is a paraneoplastic disorder associated with plasma cell dyscrasia, and that a therapeutic approach for multiple myeloma using thalidomide and dexamethasone can also be effective for treating POEMS. In addition, we investigated the feasibility of a randomised control study of POEMS syndrome and validated the assessments of the therapeutic effects.

## Methods

### Trial design

**Figure 1:**
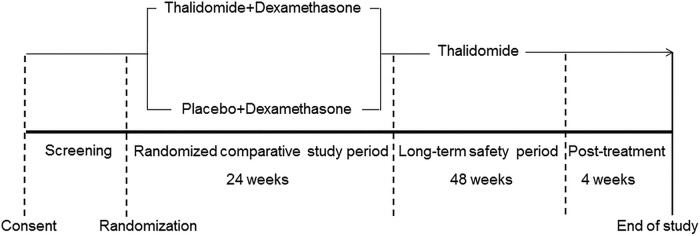
Schematic depiction of the trial design. Eligible participants are randomly assigned to a 24-week treatment of thalidomide (100–300 mg daily) plus dexamethasone (12 mg/m^2^ on days 1–4 of a 28-day cycle) or placebo plus dexamethasone (randomised comparative study period). Patients who complete the randomised comparative study period or show subacute deterioration within the first 24 weeks participate in the subsequent 48-week open-label safety study (long-term safety period).

The J-POST Trial is a 24-week multicentre, double-blinded, placebo-controlled randomised clinical trial of treatment of POEMS syndrome using thalidomide and dexamethasone (randomised comparative study period), followed by a 48-week open-label safety study (long-term safety period). Screening is undertaken within 28 days of randomisation to assess eligibility and collect baseline data. Patients who satisfy the eligibility criteria are randomly assigned (1:1) to receive thalidomide (100–300 mg daily) and dexamethasone (12 mg/m^2^ on days 1–4 of a 28-day cycle) or placebo and dexamethasone. Patients who complete the randomised comparative study period or show subacute deterioration within the first 24 weeks participate in the subsequent 48-week open-label safety study. After this, a 4-week post-treatment observation period is scheduled. The primary end point of the randomised comparative study period is centrally assessed in the full analysis set of the reduction rate in VEGF levels at 24 weeks, and that of the long-term safety period is adverse events (AEs) associated with thalidomide. A schematic depiction of the trial design is summarised in figure 1.

### Eligibility criteria

Eligible patients are those who meet all of the following inclusion criteria and who do not have any listed exclusion criteria.

*Inclusion criteria*

1. POEMS syndrome diagnosed according to published diagnostic criteria as ‘Probable’ or ‘Definite’ (box 1^20^).
2. Age ≥20 years.
3. Eastern Cooperative Oncology Group Performance Status of 0–3.
4. Overall score on the neuropathy limitation scale of 0–9.
5. Any of the following laboratory abnormalities: serum alanine aminotransferase or aspartate aminotransferase levels >4 times the normal upper limit; creatinine levels >1.5 times the normal upper limit.
6. Hospitalisation at the initiation of the randomised comparative study period and of the long-term safety period.
7. Regular clinic visits every 4 weeks.
8. No clinically significant ECG abnormality.
9. Signed written informed consent form.
10. Ineligibility for HDCT with ASCT during the study period.
11. Informed consent to thalidomide education and risk management system.

Box 1 **Diagnostic criteria of POEMS (polyneuropathy, organomegaly, endocrinopathy, M-protein and skin changes) syndrome (modified from Misawa and Kuwabara^20^)**

Major criteria

(a) Polyneuropathy

(b) Monoclonal plasma cell proliferative disorder

(c) Elevation of serum vascular endothelial growth factor levels

Minor criteria

(d) Sclerotic bone lesions

(e) Castleman disease

(f) Organomegaly (hepatosplenomegaly or lymphadenopathy)

(g) Oedema (oedema, pleural effusion or ascites)

(h) Endocrinopathy (adrenal, thyroid, pituitary, gonadal, parathyroid or pancreatic)*

(i) Skin changes (hyperpigmentation, hypertrichosis, plethora, cyanosis, haemangiomata or white nails)

(j) Papilloedema

(k) Thrombocytosis and/or polycythaemia

Definite POEMS syndrome: three major criteria and at least one minor criterion.

Probable POEMS syndrome: two major criteria, with at least one minor criterion.

*Because of the high prevalence of diabetes mellitus and thyroid abnormalities, this diagnosis alone is not sufficient to meet this minor criterion.

*Exclusion criteria*

1. Use of thalidomide, melphalan or bortezomib within 24 weeks of providing informed consent.
2. Unstable patients.
3. Oral or intravenous use of steroids within 4 weeks of providing informed consent.
4. Females who are pregnant or desire childbearing. Males who desire fertility.
5. Other serious and unstable medical conditions, such as cardiac failure, renal failure, liver failure, bleeding ulcers, ileus and uncontrolled diabetes.
6. Malignancy other than POEMS syndrome.
7. Known allergy to thalidomide or dexamethasone.
8. Serious mental disorder.
9. Use of any other experimental drug or therapy within 12 weeks of providing informed consent.
10. Use of prohibited drugs (other than β-blockers) or therapy within 4 weeks of the baseline.
11. Receiving a judgement of inappropriateness for the study.

### Recruitment

This trial was declared and registered at the Pharmaceuticals and Medical Devices Agency in September 2010. Recruitment into the trial started in November 2010 and ended in February 2014, or until a total of 24 participants had been recruited. The treatment follow-up of the participants is currently ongoing and the last visit of the last patient is due to take place in August 2015. This study is being conducted at 12 tertiary neurology centres in Japan.

### Sample size calculation

Twenty-four patients will be randomised and included in the study. This sample size was based on results from our previous studies^8^ ^13^ and the database of patients with POEMS syndrome; therefore, the estimated values of the reduction rate of serum VEGF level over 24 weeks were 0.55 (SD=0.21) after thalidomide–dexamethasone treatment and 0.35 (SD=0.20) after melphalan–prednisone treatment. Assuming a group difference of 0.35 (SD=0.25), 10 patients per arm will provide >80% power to detect a difference in the reduction rate of serum VEGF levels between thalidomide and placebo treatment for at least 24 weeks using a two-sided, two-sample t test at a 5% level of significance. Thus, to allow for a 20% dropout rate, 12 participants are required per group, for a total of 24 participants in the study.

### Allocation

A registration form for an eligible patient will be sent by the investigators to the registration centre at EPS Associates Co, Ltd (by Fax). Registration and allocation will be implemented at the registration centre. Eligible patients who provide written informed consent will be randomised to either thalidomide or placebo at a ratio of 1:1 by a computer program located at the registration centre, using a minimisation method^21^ ^22^ with biased coin assignment balancing on serum VEGF levels (≤3000 or >3000 pg/mL) and the evidence of pleural effusion (yes or no) at the screening test. The trial medication (with a unique number) will be distributed by the coordinating investigator to each hospital at the start of the trial. Investigators will prescribe the trial medication according to the number allocated at the registration centre.

### Blinding

Participants and study personnel will be blinded to thalidomide or placebo treatment until the code is opened. Placebo capsules are indistinguishable in appearance from the thalidomide capsules. Serum VEGF levels will be measured at the central laboratory (SRL Medisearch Inc, Tokyo, Japan) and will also be masked to participants and study personnel from the baseline measurement to the opening of the code.

In case of emergencies for which it becomes necessary to unmask the blinding to make an adequate treatment decision, the blinding can be lifted by the investigator if deemed necessary. Patients in whom the blinding has been lifted will be removed from the trial immediately.

### Interventions

#### Randomised comparative study period

Each treatment cycle will consist of 4 weeks (days 1–28), and thalidomide, or placebo, and dexamethasone will be administered for 24 weeks (six cycles). Thalidomide or placebo will be given on days 1–28, and dexamethasone will be administered at a dose of 12 mg/m^2^ on days 1–4. The trial medication will be initiated on the randomisation day at a dosage of one capsule containing 100 mg of thalidomide or placebo, to be administered at bedtime every 2 days. The dose will increase to one capsule daily on day 8 and two capsules daily on day 15, and participants will continue to take two capsules daily after the titration period, if there is no haematological or skin toxicity that exceeds the Common Terminology Criteria for Adverse Events (CTCAE) of grade 3. The administration of thalidomide or placebo can be decreased and then discontinued as required during the study period, in cases that exhibit development of haematological or skin toxicity that exceeds the CTCAE of grade 3, or other AEs, for which investigators assume that dose reduction is appropriate.

Patients who experience subacute worsening of POEMS syndrome with subacute capillary leak-like symptoms (ie, 5 kg/month of weight gain or pleural effusion increase) or evident deterioration of neuropathy (ie, increase in the total score on the overall neuropathy limitation scale of >2) will promptly be shifted from the randomised comparative period to the long-term safety period.

#### Long-term safety period

Each treatment cycle will consist of 4 weeks (days 1–28) and only thalidomide will be administered for 48 weeks (12 cycles). The trial medication will be initiated on the first day of the long-term safety period at a dosage of one capsule (100 mg) of thalidomide, to be administered at bedtime every 2 days. The dose will increase to one capsule daily on day 8 and two capsules daily on day 15, and participants will continue to take two capsules daily after the titration period if there is no haematological or skin toxicity that exceeds the CTCAE of grade 3. The administration of thalidomide or placebo can be decreased and then discontinued as required during the study period, if there is haematological or skin toxicity that exceeds grade 3 in the CTCAE, or other AEs, for which investigators assume that dose reduction is appropriate.

Patients who experience subacute worsening of POEMS syndrome with subacute capillary leak-like symptoms (ie, 5 kg/month of weight gain or pleural effusion increase) or evident deterioration of neuropathy (ie, increase in the total score on the overall neuropathy limitation scale >2) will be treated with three capsules of thalidomide. If patients show further deterioration, 12 mg/m^2^ of dexamethasone will be given to patients on days 1–4 of each cycle, in combination with thalidomide.

#### Treatment compliance

To evaluate treatment compliance, the number of capsules (thalidomide or placebo) remaining in each supply prescribed for patients will be counted.

#### Concomitant medication

The drugs or therapies, that is, anticancer agents other than thalidomide, radiotherapy or oral or intravenous steroids, are not permitted throughout the study.

### Outcomes

#### Randomised comparative study period

The primary outcome measure is the reduction rate of serum VEGF level over 24 weeks after treatment by mutual agreement between the Pharmaceutical and Medical Devices Agency (PMDA) and the J-POST Trial, because VEGF levels are considered as a surrogate marker used to evaluate disease activity in POEMS syndrome.^7–10^ The definition of the reduction rate is as follows: serum VEGF level reduction rate=(VEGF level at the baseline–VEGF level at 24 weeks)/VEGF level at the baseline. The secondary end points include changes in serum VEGF levels, the achievement of a normal range of serum VEGF level (<1000 pg/mL), motor functions (sum scores of manual muscle testing (MMT), grip and overall neuropathy limitation scale), parameters of nerve conduction studies (motor conduction velocity (MCV), compound muscle action potential (CMAP) amplitude and F-wave latency), M-protein levels (serum and urine), pleural effusion, vital capacity, body weight and quality of life (QOL, SF-36)^23^ ^24^ as well as AEs.

#### Long-term safety period

The primary outcome measure will be AEs, because the major aim of the long-term safety period is to investigate the safety of thalidomide administration for 12–18 months. The secondary end points include changes in serum VEGF levels, the achievement of a normal range of serum VEGF levels (1000 pg/mL), motor functions (MMT sum score, grip and overall neuropathy limitation scale), parameters of nerve conduction studies (MCV, CMAP amplitude and F-wave latency), M-protein levels (serum and urine), pleural effusion, vital capacity, body weight and QOL (SF-36).

### Data collection

#### Trial visits and examinations

The trial is divided into four periods: (1) screening; (2) randomised comparative study period (24 weeks, six cycles); (3) long-term safety study period (48 weeks, 12 cycles); and (4) post-treatment observation period. Each treatment cycle consists of 4 weeks (days 1–28), and patients will make visits on day 1 of each cycle during the study period. For all female participants of reproductive age, pregnancy tests will be conducted every 28 days. The schedule for the study visits and data collection is summarised in table 1.

**Table 1:** Schedule of data collection

	Screening	Randomised comparative study period					Long-term safety period					
		C1		C2	C3–6	EOT	C1		C2	C3–6	EOT	Follow-up 4 weeks after EOT
		D1	D8	D1	D1		D1	D8	D1	D1		
Informed consent	X											
Clinical assessment*	X	X	X	X	X	X	X	X	X	X	X	X
Vital signs†	X	X	X	X	X	X	X	X	X	X	X	X
Blood/urine tests‡	X	X	X	X	X	X	X	X	X	X	X	X
Endocrine tests (fasting)		X					X					
VEGF measurements	X	X		X	X	X	X		X	X	X	X
Chest X-ray	X	X		X	X	X	X		X	X	X	
ECG	X	X	X		X	X	X	X		X	X	
CT	X	X			X	X	X			X	X	
Nerve conduction studies		X			X	X	X			X	X	
Respiratory function tests		X				X	X				X	
SF-36		X				X	X				X	
Adverse events		X	X	X	X	X	X	X	X	X	X	X
Pregnancy tests§	X	X	X	X	X	X	X	X	X	X	X	X

*Clinical assessment: complete history/examination (screening), focused history/examination (during study period).

†Vital signs: heart rate, blood pressure, weight.

‡Blood/urine tests include free-light chain and immunofixation of M-protein on D1 of C1 and 3 of randomised, comparative study period and on D1 of C1 and 3 of long-term safety period.

§Pregnancy tests will be examined in all female participants of reproductive age every 28 days.

C, cycle; D, day; EOT, end of treatment; SF-36, MOS Short-Form 36-Item Health Survey; VEGF, vascular endothelial growth factor.

### Data management, monitoring and auditing

The investigators (or their delegates) will maintain individual records for each patient as source data, which include a log of informed consent, medical records, laboratory data and other records or notes, as appropriate. All entries in the case report form (CRF) must be backed up by the relevant source data. All source data will be kept according to good clinical practice (GCP). CRFs must be completed in a timely manner.

All data are collected by the independent data management centre that was established for the present study. There will be no direct communication between POEMS investigators and the Coordinating Data Centre. The clinical data entry (double data entry), coding, data management and reporting will be performed using the data management system CLiSSS (Medical Edge Inc, Tokyo, Japan). Data management will be carried out according to the standards of procedure of the trial.

A monitor will confirm that the investigational team is adhering to the protocol and GCP, that data are being accurately recorded in CRFs, that AEs have been properly documented on CRFs, that severe AEs (SAEs) have been forwarded to the coordinating investigator and the provider of the investigational product, and that the SAEs that met criteria for reporting have been forwarded to the Institutional Review Board (IRB). An interim analysis will not be performed.

The study may be audited or inspected by the provider of the investigational product or PMDA. In case of an audit, the investigators must make all study documentation available to the auditor. If an audit or inspection occurs, the investigators at the study site must discuss the findings and any relevant issues.

### Harms

Investigators must record all AEs in the patients’ CRFs. The National Cancer Institute's CTCAE (V.4.0) will be used to grade each AE. All AEs are to be followed up continually during their course until resolution, or for 4 weeks after the end of the trial. All SAEs must be reported to all investigators and discussed through a web-based AE reporting system; SAEs that were not reported previously will be reported to PMDA.

### Statistical methods

The analyses of the primary and secondary outcomes will be performed in the full analysis set. For the baseline variables, summary statistics will be constructed using frequencies and proportions for categorical data, and means and SDs for continuous variables. Patient characteristics will be compared using Fisher's exact test for categorical outcomes, and t tests or the Wilcoxon rank sum test for continuous variables, as appropriate.

For the primary analysis, which will be aimed at comparing treatment effects, the least squares means (LSMeans) and their 95% CI, which are estimated using analysis of covariance (ANCOVA) of the reduction rate of serum VEGF levels (untransformed) on week 24, will be compared between the thalidomide and placebo groups using an ANCOVA model, taking into account the variation caused by treatment effects and using the baseline serum VEGF levels (≤3000 or >3000 pg/mL) and evidence of pleural effusion as covariates. To compare the treatment groups, the difference in LSMeans and the 95% CIs will be expressed as a proportion of the reference treatment LSMean. The primary analyses will be performed in the full analysis set without imputing missing observations. As a sensitivity analysis, a mixed-effect model for repeated measures (MMRM) and the last observational carried forward (LOCF), and the multiple imputation methods will be applied to examine the effect of missing data. The secondary analysis will be performed in the same manner as the primary analysis.

All comparisons are planned and all p values will be two sided. p Values of <0.05 will be considered statistically significant. All statistical analyses will be performed using the SAS software V.9.3 (SAS Institute, Cary, North Carolina, USA). This statistical analysis plan was developed by the chief investigator and the statistician at Chiba University before completion of the patient recruitment and data collection.

### Ethics and dissemination

#### Research ethics approval and protocol amendments

Substantial amendments of the study protocol must be approved by IRB. The trial was notified and registered at PMDA (No. 22-1716), and at the UMIN Clinical Trials Registry (UMIN000004179) and JMACCT Clinical Trials Registry (JMA-IIA00046).

#### Informed consent

All participants will receive adequate information about the nature, purpose, possible risks and benefits of the trial, and on alternative therapeutic choices using an informed consent approved by IRB. A participant must be given ample time and opportunity to ask questions and to consider participation in the trial. A completed informed consent is required for enrolment in the trial. The investigators must maintain the original signed consent form and a copy of the signed consent form.

#### Confidentiality

To assure confidentiality, trial participants will be allocated a unique trial identification number throughout the trial.

#### Dissemination

The findings of this trial will be disseminated through peer-reviewed publications and conference presentations and will also be disseminated to participants.

## Discussion

The J-POST Trial is the first randomised control trial (RCT) to investigate the efficacy and safety of a therapeutic agent for POEMS syndrome. RCTs are essential to establish quality evidence, although it is generally difficult to conduct RCTs for rare and severe diseases, such as POEMS syndrome, from the viewpoints of designing appropriate study schema and recruiting patients. This trial can be a prototype RCT for POEMS syndrome and contribute considerably to the future evolution of treatment for this syndrome.

The application of therapeutic interventions for multiple myeloma to POEMS syndrome has quite improved its prognosis.^15^ ^20^ In the near future, the number of new therapeutic choices for multiple myeloma, such as next-generation immunomodulatory drugs, proteasome inhibitors, signal transduction inhibitors and molecular targeted drugs, will be available and may be effective for POEMS syndrome.^20^ Prospective clinical trials are vital to establish evidence-based treatment strategies for the management of the increasing therapeutic choices. Moreover, RCTs are optimal to prove the efficacy and safety of each agent, if possible.

There were some limitations to this study. First, the natural history of POEMS syndrome remains to be elucidated. Patients with POEMS syndrome generally show subacute deterioration and cannot walk independently within 1 year of the onset of the disease.^25^ Conversely, in some patients, the disease progresses very slowly. At present, we cannot foresee disease courses exactly at the initial diagnosis of a patient. Recruiting patients with various disease courses into the trial can affect the results substantially. To avoid the recruitment of patients with specific disease course into either the thalidomide or placebo group, randomisation will be stratified according to VEGF levels, which can reflect disease activity, and pleural effusion, which can sometimes be life-threatening in POEMS syndrome.

The second limitation was that this trial employed a surrogate marker, instead of a hard end point, that is, the reduction rate of serum VEGF level over 24 weeks after treatment, as the primary outcome. Markedly elevated serum VEGF levels are specifically found in patients with POEMS syndrome,^3^ ^5^ ^6^ and the characteristic features of this syndrome (eg, pleural effusion, oedema or angiomata) are consistent with the physiological effects of VEGF, such as increased vascular permeability and angiogenesis.^26^ VEGF levels generally decrease in response to treatment and are considered to reflect disease activity.^7–10^ In this study, we will also prospectively investigate changes in clinical observations and laboratory parameters over 18 months, to test the adequacy of serum VEGF levels as a surrogate end point.

Close observational studies and an appropriate rationale are essential for good-quality prospective clinical trials, and enable the conduct of RCTs even in rare and fatal diseases. This study may be a major turning point in the therapeutic approach for POEMS syndrome, as well as a model to establish evidence in rare diseases.

## Supplementary Material

Reviewer comments
