# Supplementary material for: Japanese POEMS syndrome with Thalidomide (J-POST) Trial: study protocol for a phase II/III multicentre, randomised, double-blind, placebo-controlled trial
Source: BMJ Open. 2015 Jan 8;5(1):e007330. doi: 10.1136/bmjopen-2014-007330 (PMC4289734; doi:10.1136/bmjopen-2014-007330)
Supplement: Reviewer comments [file bmjopen-2014-007330.reviewer_comments.pdf]

PEER REVIEW HISTORY

BMJ Open publishes all reviews undertaken for accepted manuscripts. Reviewers are asked to complete a checklist review form (**http://bmjopen.bmj.com/site/about/resources/checklist.pdf**) and are provided with free text boxes to elaborate on their assessment. These free text comments are reproduced below.

ARTICLE DETAILS

**TITLE (PROVISIONAL)**	Japanese POEMS syndrome with Thalidomide (J-POST) Trial: study protocol for a phase II/III multi-centre, randomized, double-blind, placebo-controlled trial
**AUTHORS**	Katayama, Kanako; Misawa, Sonoko; Sato, Yasunori; Sobue, Gen; Yabe, Ichiro; Watanabe, Osamu; Nishizawa, Masatoyo; Kusunoki, Susumu; Kikuchi, Seiji; Nakashima, Ichiro; Ikeda, Shu-ichi; Kohara, Nobuo; Kanda, Takashi; Kira, Jun-ichi; Hanaoka, Hideki; Kuwabara, Satoshi

This article was not externally reviewed at BMJ Open. Protocols that have been independently assessed prior to submission to BMJ Open are usually fast-tracked to publication on the grounds that further substantial changes will not be possible. This independent assessment will usually be external, independent review for both a substantial grant award from a non-commercial or government funder and ethics approval.
